# Type of arrhythmias and the risk of sudden cardiac death in dialysis patients: a systematic review and meta-analysis

**DOI:** 10.1186/s43044-025-00606-6

**Published:** 2025-01-13

**Authors:** Subhash Chander, Ahmad Bin Aamir, Rabia Latif, Om Parkash, F. N. U. Sorath, Sam Tan, Abhi Chand Lohana, Sheena Shiwlani, Mohammed Yaqub Nadeem

**Affiliations:** 1 Department of Critical Care Medicine, Mayo Clinic, Rochester, MN USA; 2 Department of Medicine, Faisalabad Medical University, Faisalabad, Pakistan; 3 Department of Medicine, Montefiore Medical Center, Bronx, NY USA; 4 Department of Anesthesiology, Dow Health Science, Karachi, Pakistan; 5 Department of Medicine, Icahn School of Medicine, New York, NY USA; 6 Department of Medicine, Western Michigan University, Kalamazoo, WV USA; 7 Department of Physiology, College of Medicine, Imam Abdulrahman, Bin Faisal University, Dammam, Saudi Arabia

**Keywords:** Sudden cardiac death, Arrhythmia, Bradyarrhythmia, ESRD, Hemodialysis

## Abstract

**Background:**

Patients on long-term dialysis for end-stage kidney disease have a high mortality rate, predominantly due to sudden cardiac death (SCD), which is associated with an increased risk of arrhythmias compared to the general population. Thus, the current systematic review and meta-analysis aimed to investigate the incidence of SCD among dialysis patients at risk of arrhythmia.

**Methods:**

This systematic review and meta-analysis followed the PRISMA guidelines. PubMed, Cochrane Library, Google Scholar, Medline, and Europe PMC were searched for articles meeting our inclusion criteria. Studies with risk assessment of arrhythmias and the incidence of SCD in dialysis patients were considered for inclusion. Effect size from eligible studies was pooled using a random effects model and restricted maximum likelihood estimation. Heterogeneity was quantified using the *I*^2^ statistic, and the risk of publication bias was evaluated by visually inspecting funnel plots.

**Results:**

Our search strategy yielded 5861 studies, of which 1960 duplicate entries were removed in the prescreening stage, 3326 were excluded after title/abstract screening, and 519 after full-text screening for not meeting our inclusion criteria. Finally, 11 studies were included in the analysis, and two more were selected from the bibliography list of previous reviews. Eight included studies were randomized controlled trials, and five were cohort studies, which provided a pooled population size of 12,611 dialysis patients for the meta-analysis, which indicated a significantly larger effect size of arrhythmia [Cohen’s *d* = 110.38 (95%CI 42.72–178.05), *p* = 0.0]. Visual assessment of the funnel plot indicated no publication bias.

**Conclusion:**

SCD remains a significant public health concern, particularly in patients undergoing dialysis. Meta-analysis results show that bradyarrhythmia is a common arrhythmic condition leading to SCD; however, other arrhythmias should also be considered.

## Background

Patients undergoing dialysis have a high prevalence of cardiovascular disease (CVD), which is associated with an increased risk of arrhythmias compared to the general population. According to the United States Renal Data System for 2018, patients on maintenance dialysis have a greater mortality risk than patients with preserved kidney function, with mortality rates exceeding 180 per 1000 patient-years [1]. A significant cause of mortality in such patients is sudden cardiac death (SCD), which is defined as an unexpected death from cardiac causes in a person with a known or undiagnosed cardiac condition that occurs within one hour of the onset of symptoms (witnessed SCD) or within twenty-four hours of the last sign of life (unwitnessed SCD) [2]. However, it is challenging to determine the prevalence of SCD precisely as its incidence among patients with end-stage kidney disease (ESKD) is frequently mixed with the incidence of sudden cardiac arrest (SCA) that occurs during dialysis sessions even though extra dialysis SCD and intradialysis SCA reflect different clinical scenarios [2]. Nevertheless, current data suggests that SCD is more common among hemodialysis patients in the United States, accounting for 33% of all deaths than in other countries such as Japan (23%), Australia (19%), and Canada (18%) [3].

Arrhythmias are a major contributor to the risk of SCD. About 40% of deaths in hemodialysis patients with a known cause are attributable to SCD and arrhythmia [4]. In comparison, SCD accounts for up to 15% of all deaths in the general population. Some studies have shown a correlation between the timing of dialysis sessions and episodes of SCD, as well as relationships with serum or dialysate electrotype concentrations, which strongly indicate arrhythmia as a primary cause of SCD during dialysis [4]. Some existing research studies have identified the differences between SCD among dialysis patients and normal individuals. While the combination of a vulnerable myocardium and acute proarrhythmic trigger leads to terminal arrhythmias in dialysis patients, this condition manifests as ischemic cardiomyopathy with a reduced left ventricular ejection fraction (LVEF) in the general public, which typically leads to disorganized cardiac conduction [5, 6]. The intermittent treatment pattern in patients with ESKD receiving dialysis further exacerbates the risk of SCD gradually with the duration of treatment by causing volume and electrolyte balance fluctuations [7].

Moreover, different arrhythmias may not equally contribute to the risk of SCD in dialysis patients. For instance, it is possible that occult myocardial ischemia, intradialytic hypotension, and post-dialysis metabolic alterations, including hypokalemia, hypocalcemia, and metabolic alkalosis, may contribute to the higher prevalence of ventricular tachycardia and ventricular fibrillation in the early stages of dialysis [7]. However, a causal relationship between arrhythmias and SCD in dialysis patients is yet to be established. Thus, the underlying causes of SCD in dialysis are still debated, especially the precise type of terminal arrhythmia, which has important implications for prevention.

In addition, Dalal et al. [8] confirmed that severe ischemic cardiomyopathy and advanced chronic kidney disease increase the incidence of ventricular arrhythmias. For instance, patients with stage 3 chronic kidney disease had a six-fold increased risk of developing ventricular fibrillation following a first myocardial infarction. In addition, advanced chronic kidney disease is a strong predictor of appropriate shock therapy delivery for ventricular arrhythmias among implantable cardioverter-defibrillator recipients. However, retrospective studies in this high-risk population did not show any survival improvement after the implantation of cardiovascular defibrillation [7]. Moreover, it remains unclear what causes SCD in dialysis patients with preserved left ventricular function. Recent studies have found higher rates of bradyarrhythmic episodes and mortality in small cohorts of asymptomatic dialysis patients using implantable loop recorders (ILRs) [4]. Further, dialysis patients have a significantly elevated atrial fibrillation and stroke risk [9]. Atrial fibrillation affects 20–50% of patients, according to claims data, although the exact prevalence is probably much greater because most episodes are asymptomatic. Stroke risk is 20% in patients starting dialysis, and more than half of these strokes are classified as cardioembolic or cryptogenic, with atrial fibrillation being a potential underlying cause in these cases [7].

Thus, it is essential to determine whether and how frequently dialysis causes potentially fatal arrhythmias to optimize clinical outcomes among patients undergoing dialysis. This includes identifying the arrhythmic forms most commonly associated with SCD, particularly terminal rhythms. The need for cardiac monitoring is driven by the documentation of premature ventricular or atrial contractions, peridialytic changes in electrocardiographic morphology, and changes in heart rate variability. However, until recently, surface electrocardiography (ECG) or Holter monitoring technology restricted the realistic monitoring duration to periods too short to accurately capture the occurrence of arrhythmia or sudden cardiac death [4]. Although ILRs may help identify terminal arrhythmias, a coordinated effort would be required given the low enrollment rates anticipated in such studies, as underscored by the kidney disease improving global outcomes (KDIGO) clinical update conference on cardiovascular disease in chronic kidney disease nearly 5 years ago [10]. In addition to diagnosing fatal arrhythmias, ILRs can also help in the detection of bradyarrhythmia and atrial fibrillation. The Monitoring in Dialysis Trial (MiD), which employed ILR to identify arrhythmia in the context of HD, was the source of data on arrhythmia in dialysis patients reviewed in this publication [11].

Uncertainty around the “terminal event,” particularly in distinguishing sudden death from an arrhythmia from sudden death that cardiac devices cannot prevent, constitutes a significant knowledge gap in our understanding of sudden cardiac events in dialysis patients. The KDIGO identified the lack of autopsy data on dialysis-related SCD as a significant knowledge gap, while underscoring the limitations of traditional definitions, which include “sudden, unexpected death within an hour of symptom onset, or unwitnessed, unexpected death without obvious non-cardiac cause in patients known to be well within the past 24 h.” In a population with a high prevalence of comorbid illness who spend a disproportionate amount of time in healthcare facilities, what exactly is “unexpected death”? Patients eventually die of terminal arrhythmias after discontinuing dialysis, although this is a withdrawal-related death. Without a patient-centered context, it would be simple to deduce incorrectly from an ILR that SCD was the “primary” event. Similarly, in the absence of rhythm tracings, conditions such as subarachnoid hemorrhage or aortic dissection may resemble SCD [11].

Although cardiovascular diseases and SCD are now generally acknowledged as significant risk factors for poor outcomes in dialysis patients, conventional cardiovascular risk factors do not adequately explain this association [12]. Although chronic kidney disease and coronary artery disease frequently coexist, sudden cardiac death is also widespread in dialysis patients with no history of coronary artery disease or compromised left ventricular ejection fraction. It is possible that endothelial dysfunction, electrolyte fluxes, chronic inflammation, insulin resistance, and autonomic instability, as well as the resulting bone mineral abnormalities and vascular calcification, increase the cardiovascular risk associated with severe chronic kidney disease [7].

Therefore, the relationship between chronic kidney illness and arrhythmias remains poorly understood. In addition, there is an urgent and unmet clinical need for sophisticated risk stratification strategies to identify dialysis patients who would most benefit from ICD implantation in the dialysis population, where severe arrhythmic risk has already been established [11]. Thus, depending on data availability, the current systematic review and meta-analysis aim to investigate the types of arrhythmias and the incidence of SCD among dialysis patients.

## Method

### Design

The preferred reporting items for systematic review and meta-analyses (PRISMA) guidelines were implemented using the Cochrane technique [13]. In addition, the inclusion and exclusion criteria were developed using the populations, interventions, comparators, and outcomes (PICO) framework. The study was prospectively registered with PROSPERO (International Prospective Register of Systematic Review) under CRD42023411441.

### Search strategy

Digital databases (PubMed, Cochrane Library, Google Scholar, Medline, and Europe PMC) and the bibliography of earlier systematic reviews and meta-analyses were searched for relevant articles. Database searches were performed using the following keywords and Medical Subject Headings (MeSH): arrhythmias, sudden cardiac death, dialysis, hemodialysis, end-stage renal disease, coronary artery disease, cause of death, death, sudden cardiac arrest, SCD, coronary artery disease (cad), sudden cardiac arrest, life-threatening arrhythmia, and cardiac arrhythmia. These keywords and MeSH phrases were derived through a computerized search of PubMed, Medline, and Europe PMC to help create reliable search strings. To obtain the references most pertinent to our search inquiry, we developed a search strategy that used Boolean operators like (AND) and (OR). Field tags (tabs and two) were also incorporated to generate variations in the search string for study identification.

### Eligibility criteria

The reviewers evaluated the retrieved papers for eligibility using predefined inclusion and exclusion criteria. Articles retrieved from PubMed, Medline, and Europe PMC were inclusive of the following data: a population consisting of any ages, recent publications in the English language, only human participants, free, full-text papers, and study reports from years not preceding 5 years. The time filter ensured that only the latest evidence was included in the current systematic review and meta-analysis.

A study was considered for inclusion if it provided the intended result (risk assessment of arrhythmias, rate of SCD in the dialysis population, and prevention of such). The following study designs were eligible for inclusion: cohort studies, randomized controlled trials, clinical trials, and comparative studies comparing sudden cardiac mortality rates in dialysis and general cardiac-related illness populations. Studies considered for inclusion had to have published work on the risk factors that are prone to cardiovascular complications in dialysis patients. Findings on the development of cardiovascular complications among various age brackets of a given population should be available in the retrieved studies. Single-out high-risk patients undergoing dialysis or hemodialysis treatment with fatal, life-threatening cardiovascular conditions were also considered for inclusion. The results of the independent eligibility assessments were combined and corrected, taking into account inputs from other parties.

### Screening and data extraction

The reviewers based the initial screening of data on titles and abstracts and the subsequent full-text screening of the included articles. Study characteristics (author and publication year), place of study, study design, population, age, sex, arrhythmia events, SCD mortality, risk of SCD, and conclusion were extracted from the eligible studies using a coded Microsoft Excel sheet.

### Statistical analysis

Review Manager version 5.4 (RevMan 5.4: The Nordic Cochrane Center, The Cochrane Collaboration, 2014) was used for the current systematic review and meta-analysis, including generating funnel and forest plots. The risk of arrhythmia in dialysis patients was assessed by calculating effect size and respective 95% confidence intervals (CI). Heterogeneity between the studies was statistically quantified using the *I*^2^ statistic. Heterogeneity was categorized as low (25%), moderate (50%), or high (75%) based on previous studies by Higgins et al. [14]. Effect size from eligible studies was pooled using a random effects model and restricted maximum likelihood estimation (REML) to calculate pooled effect size and heterogeneity iteratively [15]. Publication bias was evaluated by visually inspecting funnel plots.

## Results

### Study selection

**Fig. 1:**
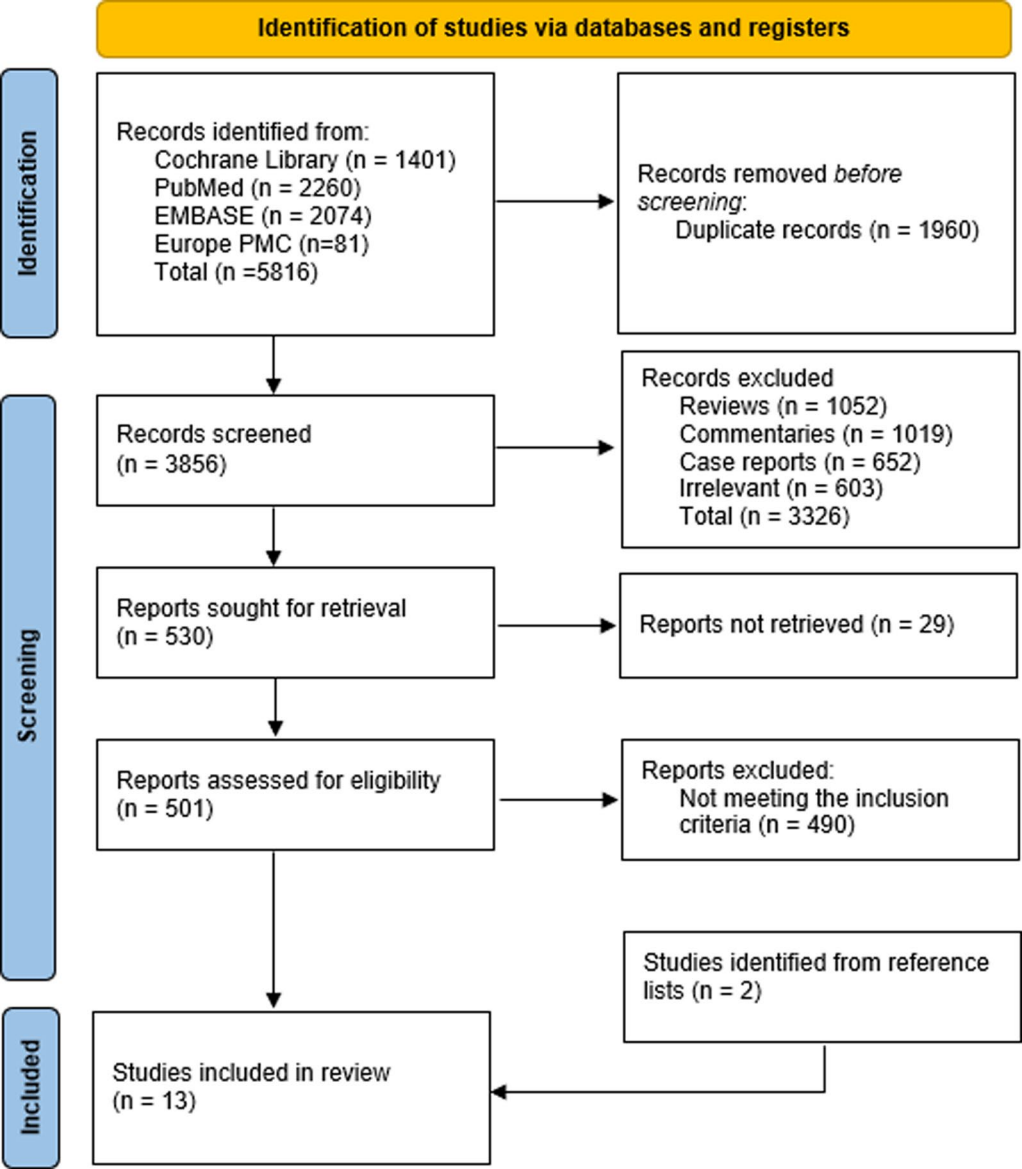
PRISMA flow diagram for the study selection for the systematic review

The initial search generated 5816 studies, of which 1960 entries were removed due to duplication. The remaining 3856 articles were subjected to title and abstract screening, where 3326 studies were excluded for not meeting our inclusion criteria. Full-text screening was performed for 530 studies to eliminate 519 additional studies. Finally, 11 studies were included in the analysis, and two more were selected from the bibliography list of previous reviews, bringing the final tally to 13 articles used for data extraction [16–28]. Among the included studies, eight were randomized controlled trials [16–18, 21, 23, 25, 27, 28], and five were cohort studies [19, 20, 22, 24, 26] (Table 1). The PRISMA 2020 flow diagram for updated systematic reviews summarizing the selection process is shown in Fig. 1.

**Table 1:** Study characteristics

Author (Year)	Design	Sample size	Age (years)	Gender (M/F)	SCD mortality	SCD risk (HR; 95% CI)	Conclusion
Pfau et al. [21]	RCT	1108	65.6 ± 8.5–67.6 ± 8.1	603/505	139	1.62; 1.03–2.56	Elevated serum oxalate represents a new risk factor for cardiovascular problems and SCD among dialysis recipients
Jukema et al. [18]	RCT	188	55–80	188/0	19	1.32; 0.53–3.29	Prophylactic ICD therapy did not lower the rate of SCD or all-cause mortality in a cohort that was well-screened and given appropriate care, while receiving dialysis
Tangri et al. [25]	RCT	1747	58	751/996	181	0.65; 0.42–1.01 (with IHD) 1.61; 0.92–2.80 (without IHD)	The use of β-blockers did not decrease the incidence of SCD in HD patients without a history of IHD
Cheung et al. [16]	RCT	1846	18–80	808/1038	343	2.57; 1.73–3.83	IHD was a significant contributor to cardiac hospitalizations and cardiac deaths
Karnik et al. [19]	Cohort Study	400	Mean = 66.3 ± 12.9 in the cardiac arrest and 60.2 ± 15.4 in the FMCNA group	206/194	14	HR not reported. Mortality rate was 60%	Cardiac arrest was a relatively rare yet serious side effect of HD. Continuous review and change of dialysate prescriptions were recommended for high-risk patients
Wang et al. [26]	Cohort Study	230	Mean = 56 ± 11	117/113	28	78.6%; 72.2–84.1%	N-terminal probrain natriuretic peptide may be utilized to identify patients at risk of SCD instead of echocardiography, although it offers no additional predictive advantage
Wanner et al. [27]	RCT	1255	18–80	0/1255	160	0.8; 0.64–1.03	Atorvastatin did not impact the composite primary endpoint of cardiovascular death, nonfatal myocardial infarction, and stroke in patients with diabetes receiving HD
Drechsler et al. [17]	RCT	1255	18–80	671/584	160	3.1; 2.0–4.9 and 2.4; 1.5–3.9	Low homoarginine levels in dialysis patients is a significant risk factor for SCD and heart failure-related mortality
Kerns et al. [20]	Cohort Study	558	≥ 18 (Mean = 56)	312/246	30	3.62; 1.36–9.66	Patients with obstructive sleep apnea receiving HD were more likely to die from all causes, have cardiovascular disease, and develop SCD
Silva et al. [24]	Cohort Study	100	Mean = 59 ± 8.8	65/35	7	2.83; 1.01–7.96	ILR helped identify a significant prevalence of arrhythmic events, the majority of which lacked clinical value, in the medium-term follow-up of renal transplant candidates
Sacher et al. [23]	RCT	71	Mean = 65 ± 9	52/21	4	46.23; 7.96–268.48	To enable early detection and the start of appropriate treatment, ILR may be considered in HD patients at risk for serious conduction abnormalities, ventricular arrhythmia, atrial fibrillation, or flutter
Wheeler et al. [28]s	RCT	3883	≥ 18	2305/1578	348	0.79; 0.64–0.98	Any advantages of cinacalcet for treating cardiovascular disease in HD may come from the reduction of nonatherosclerotic processes
Roberts et al. [22]	Cohort Study	30	Mean = 68 ± 12	18/12	2	0.87; 0.66–1.13	Bradyarrhythmia was a frequent and possibly serious arrhythmic event, confirming the high mortality observed in HD patients

FMCNA, Fresenius Medical Care North America; HD, hemodialysis; ICD, Ischemic heart disease; ILR, implantable loop recorder; RCT, randomized controlled trials; SCD, sudden cardiac death

### Meta-analysis

**Fig. 2:**
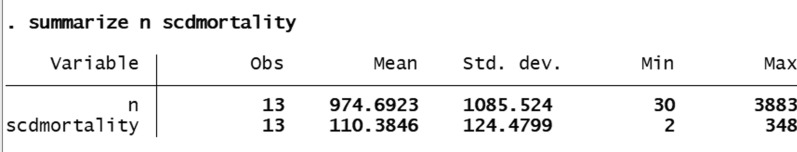
Summary statistics of the dataset calculating the standard deviation

**Fig. 3:**
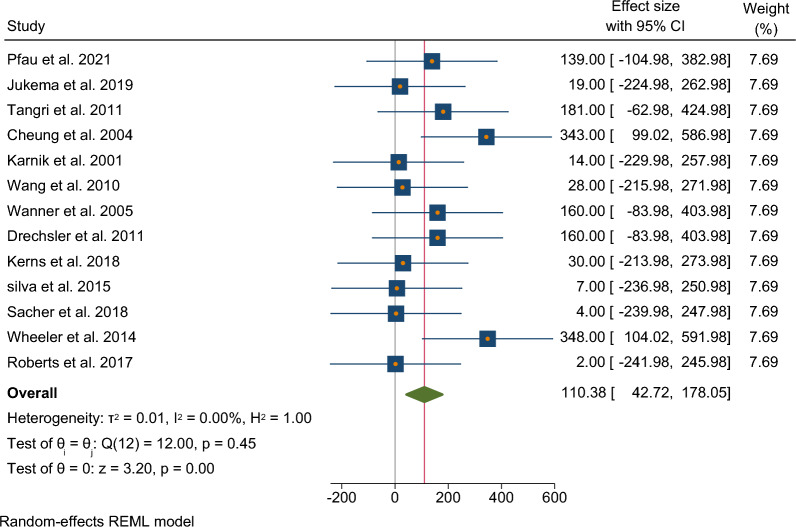
A forest plot for the meta-analysis of the incidence of sudden cardiac death in patients undergoing dialysis

**Fig. 4:**
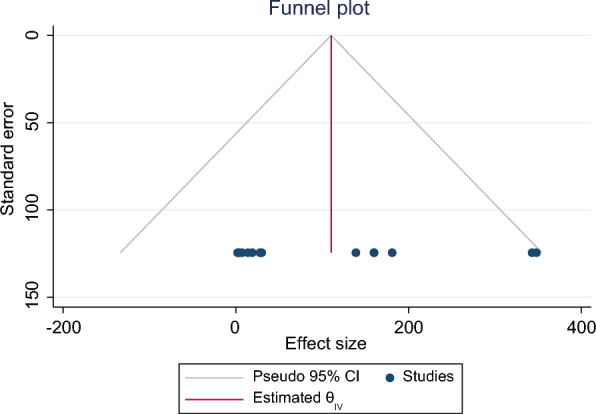
Funnel plot showing publication bias between the analyzed studies

Thirteen studies that reported the incidence of SCD mortality were included in this meta-analysis, with a pooled participant size of 12,611. The summary statistics of the dataset generated a standard deviation of 124.4799 (Fig. 2). A meta-analysis was then performed to assess the incidence of arrhythmias in patients undergoing dialysis using a random effects REML model, as no heterogeneity was detected among the 13 included studies (*I*^2^ = 0%). Analysis of the pooled data indicated a significantly larger effect size of SCD incidence [Cohen’s *d* = 110.38 (95%CI 42.72–178.05), *p* = 0.0] among dialysis patients (Fig. 3). Visual assessment of funnel plot asymmetry indicated no publication bias (Fig. 4).

## Discussion

Meta-analysis of pooled data from 13 cohort studies and randomized controlled trials showed that SCD commonly occurs in patients undergoing dialysis, confirming the findings of previous studies that patients on long-term dialysis therapy due to ESKD tend to have a high mortality rate, predominantly due to cardiovascular complications [20, 24,  and 28]. For instance, in the CRASH-ILR study, Roberts et al. [22] monitored arrhythmia and SCD in dialysis patients and reported eight deaths in 379,512 h. The study reported a 95% confidence interval for median event-free survival for any type of arrhythmia. Sacher et al. [23] also indicated that cardiac arrest accounted for nearly one-third of all cardiac mortality rates in this group of patients. These findings suggest that the risk of SCD is primarily due to the high occurrence of arrhythmias in dialysis patients, which is the leading cause of SCD [23].

Bradyarrhythmia and tachyarrhythmia are the most common rhythm abnormalities causing cardiac arrest in a dialysis patient. These outcomes are echoed by a clinical study by Tangri et al. [25], who reported that an episode of a paused heart or bradycardia mostly precedes 50% of cardiac deaths. Furthermore, Pfau et al. [21] emphasized this hypothesis with the findings that the majority of patients received a pacemaker or a defibrillating device following recommendations to do so to improve their conditions. However, all patients experience arrhythmia as the final event, which may be either ventricular fibrillation or asystole before death occurs. Such cases were observed and reported in approximately ten people who had progressive asystole or bradycardia. Previous meta-analytic studies by Roberts et al. [4] that included patients undergoing hemodialysis with long-term monitoring using ILRs illustrated that bradyarrhythmia significantly contributes to sudden death in these patients. The analysis results suggest that fatal or serious arrhythmias in dialysis patients represent defects such as asystole or bradycardia rather than tachyarrhythmias. However, dialysis patients with high cardiovascular complications who are at a greater risk of tachyarrhythmias were unrepresented in most of the studies included by Roberts et al. [4]. This problem also persisted in the current meta-analysis.

The prevalence and severity of cardiac arrest risk factors and stressors in dialysis patients are unique to dialysis sessions and result in half of all hemodialysis-related deaths caused by cardiovascular complications. Multiple studies have proposed the factors that increase the risk of cardiac arrest, complications, and death, including inflammation, hypertension, hypercoagulability, and autonomic dysfunction [16, 17, 27]. While a case-cohort study by Wang et al. [26] illustrated that patients who experienced inpatient cardiac arrest were older and had diabetes, previous studies have failed to establish any association between race or gender and SCD. However, men seem to have an increased risk of ischemic heart disease compared to women [29]. In addition, our systematic review also indicated that systolic dysfunction was the most significant predictor of SCD, followed by either low diastolic or high systolic blood pressure. For example, Karnik et al. [19] showed that reduced ventricular function predicts SCD risk. Furthermore, Jukema et al. [18] showed that poor systolic function predisposes patients to heart failure, which can increase the risk of ventricular arrhythmia through neurohumoral activation [30].

Notably, different risk factors have been observed for different types of arrhythmias in dialysis patients. For instance, Rantanen et al. [31] reported that increasing age and history of palpitations were associated with higher odds, while diabetes mellitus was associated with lower odds of paroxysmal supraventricular tachycardia in dialysis patients. In contrast, an increase in systolic blood pressure was associated with higher lower odds, while male sex was associated with higher odds of atrial fibrillation [31]. Further, increased plasma ionized calcium was associated with lower odds of non-sustained ventricular tachycardia, while bradyarrhythmia was not associated with any of the 11 independent variables used in the analysis. Only increasing age and male sex were associated with elevated odds of clinically significant arrhythmias comprising atrial fibrillation, non-sustained ventricular tachycardia, bradycardia, pauses > 3.0 s, and advanced 2nd or 3rd-degree atrioventricular block [31]. While the underlying mechanisms of dialysis triggering different types of arrhythmias are yet to be elucidated, these results strongly indicate the involvement of unique physiological processes for different types of arrhythmias.

In terms of SCD prevention in dialysis patients, Pfau et al. [21], Cheung et al. [16], and Drechsler et al. [17] suggest a common-sense approach of first identifying and then treating high-risk populations using the existing cardiovascular medications and reducing potential triggers to arrhythmic events. Tangri et al. [25] pointed out that strategies to minimize such triggers entail extended or more frequent dialysis sessions, avoiding potassium consumption, reducing dialysate temperatures, and monitoring potassium levels before dialysis sessions. Further, Wanner et al. [27] recommended other therapies to manage SCD, including anti-athymic pharmacological agents, implantable cardiac defibrillators (ICD), and pacemakers. However, these devices should be applied on a case-by-case basis because of the existing hazards that they might present to dialysis patients.

### Strength and limitations

Our pooled study population had several predisposing factors that put them at a high-risk of arrhythmia. Multiple studies have shown that arrhythmias occur in all age groups; however, the fatality rate is higher in dialysis patients due to cardiovascular complications. Therefore, the meta-analysis focused on the risk assessment of different types of arrhythmias to evaluate the mortality rate resulting from cardiac deaths in dialysis patients. Experienced researchers performed data extraction and analysis for the current study to produce high-quality information on the study topic. This was further aided by a low *I*^2^ value, indicating a high consistency among the included studies and that most of the observed variability is due to chance rather than actual differences in study outcomes [32]. Thus, our findings are robust and reliable.

However, our study comes with several limitations. Publication bias, selection bias, and the risk of over interpreting the results, especially when combining studies with different methodologies and populations, are inherent to any meta-analytic studies. In addition, our analysis was restricted to the data available in the eligible studies. For instance, we could not conduct the risk of SCD with different types of arrhythmias due to insufficient data in the available literature. In addition, the reported incidence of SCD due to arrhythmia in dialysis patients varies widely, while studies focusing only on adults or older people are scarce. Another limitation arises during multiple statistical comparisons of influences between randomized controlled studies; thus, interpretation of the additional analysis outcomes should be cautiously performed to avoid data misinterpretation.

### Study implications and future perspectives

Our meta-analysis emphasizes the need for further clinical studies to determine the incidence of SCD in dialysis patients, which remains a significant health concern. These clinical studies should focus on developing risk stratification strategies for bradyarrhythmia and tachyarrhythmia. In particular, it is important to identify the specific type of arrhythmic event that may cause SCD in dialysis patients. Thus, more randomized controlled trials with ILRs are warranted. In addition, further clinical studies should be conducted on devices with monitored and combined defibrillation and pacing capacity, owing to insufficient information on the efficacy of ICDs. Finally, mechanistic studies are needed to understand the underlying mechanisms of how dialysis triggers different types of arrhythmias and if the mechanism is related to patient characteristics.

## Conclusion

This meta-analytic study indicated a high incidence of SCD in patients undergoing dialysis. Therefore, SCD remains a major public health concern, particularly in patients undergoing dialysis, as there is a high-risk of cardiac arrest, which has the highest mortality rate among this group. Bradyarrhythmia is emerging as a common type of arrhythmia leading to SCD in the literature; however, other types should also be considered. Given the potential to improve cardiac outcomes, additional strategies should be developed to minimize cardiac mortality and morbidity in managing the dialysis population. In addition, it is essential to note the significance of the early detection and treatment of cardiovascular complications to reduce fatality rates.

## Data Availability

Data sharing is not applicable to this article as no datasets were generated or analyzed during the current study.

## Abbreviations

AF: Atrial fibrillation
CKD: Chronic kidney disease
CVD: Cardiovascular disease
ECG: Electrocardiography
ESKD: End-stage kidney disease
HD: Hemodialysis
ICD: Implantable cardioverter-defibrillator
ILR: Implantable loop recorder
OSA: Obstructive sleep apnea
PRISMA: Preferred reporting items for systematic reviews and meta-analyses
RCT: Randomized controlled trial
SCA: Sudden cardiac arrest
SCD: Sudden cardiac death
